# Association of Adiposity Indices with Platelet Distribution Width and Mean Platelet Volume in Chinese Adults

**DOI:** 10.1371/journal.pone.0129677

**Published:** 2015-06-09

**Authors:** Jian Hou, Chuanyao Liu, Ping Yao, Weihong Chen, Meian He, Youjie Wang, Yuan Liang, Xiaoping Miao, Sheng Wei, Tian Xu, Weimin Fang, Jiang Zhu, Xiulou Li, Frank B. Hu, Tangchun Wu, Handong Yang, Jing Yuan

**Affiliations:** 1 Department of Occupational and Environmental Health, School of Public Health, Tongji Medical College, Huazhong University of Science and Technology, Wuhan 430030, Hubei, P.R.China; 2 MOE Key Lab of Environment and Health, School of Public Health, Tongji Medical College, Huazhong University of Science and Technology, Wuhan 430030, Hubei, P.R.China; 3 Department of Nutrition and Food Hygiene, School of Public Health, Tongji Medical College, Huazhong University of Science and Technology, Wuhan 430030, Hubei, P.R.China; 4 Department of Maternal and Child Health, School of Public Health, Tongji Medical College, Huazhong University of Science and Technology, Wuhan 430030, Hubei, P.R.China; 5 Department of Social Medicine and Health Management, School of Public Health, Tongji Medical College, Huazhong University of Science and Technology, Wuhan 430030, Hubei, P.R.China; 6 Department of Epidemiology and Biostatistics, School of Public Health, Tongji Medical College, Huazhong University of Science and Technology, Wuhan 430030, Hubei, P.R.China; 7 Physical Examination Center, Dongfeng Central Hospital, Dongfeng Motor Corporation, Shiyan, Hubei, 442008, Hubei, P.R.China; 8 Department of Clinic Teaching and Science, Dongfeng Central Hospital, Dongfeng Motor Corporation, Shiyan, Hubei, 442008, Hubei, P.R.China; 9 Department of Nutrition and Epidemiology, Harvard School of Public Health, Boston, 02115, Massachusetts, United States of America; 10 Department of Cardiology, Dongfeng Central Hospital, Dongfeng Motor Corporation, Shiyan 442008, Hubei, China, P.R.China; School of Public Health, Zhejiang University, CHINA

## Abstract

Hypoxia is a prominent characteristic of inflammatory tissue lesions. It can affect platelet function. While mean platelet volume (MPV) and platelet distribution width (PDW) are sample platelet indices, they may reflect subcinical platelet activation. To investigated associations between adiposity indices and platelet indices, 17327 eligible individuals (7677 males and 9650 females) from the Dongfeng-Tongji Cohort Study (DFTJ-Cohort Study, n=27009) were included in this study, except for 9682 individuals with missing data on demographical, lifestyle, physical indicators and diseases relative to PDW and MPV. Associations between adiposity indices including waist circumstance (WC), waist-to-height ratio (WHtR), body mass index (BMI), and MPV or PDW in the participants were analyzed using multiple logistic regressions. There were significantly negative associations between abnormal PDW and WC or WHtR for both sexes (*p*_trend_<0.001 for all), as well as abnormal MPV and WC or WHtR among female participants *(p*_trend_<0.05 for all). In the highest BMI groups, only females with low MPV or PDW were at greater risk for having low MPV (OR=1.33, 95% CI=1.10, 1.62 *p*_trend_<0.001) or PDW (OR=1.34, 95% CI=1.14, 1.58, *p*_trend_<0.001) than those who had low MPV or PDW in the corresponding lowest BMI group. The change of PDW seems more sensitive than MPV to oxidative stress and hypoxia. Associations between reduced PDW and MPV values and WC, WHtR and BMI values in Chinese female adults may help us to further investigate early changes in human body.

## Introduction

Mean platelet volume (MPV) and platelet distribution width (PDW) are quantitative measures of the variability in platelet size. They can be used in the assessment of platelet function [1]. Numerous studies indicated that low PDW and MPV were associated with a range of diseases, including mild cognitive impairment, vascular dementia, Alzheimer’s disease, pulmonary arterial hypertension and osteoporosis [2–5]. But high PDW and MPV were related to acute myocardial infarction, unstable angina and vascular complications of diabetes [6–8]. Additionally, low PDW may be a potential indicator for individuals with a high risk of mortality and 1-year mortality in the elderly population [9]. A recent study suggested that platelet hyper-activation could accelerate atherothrombosis which may be a result of the interaction among the features clustering in obesity and metabolic syndrome [10]. However, no association was found between coronary artery disease and PDW or MPV, which may be due to the extent of coronary artery disease in response to PDW and MPV levels [11].

Overweight and obesity are defined as abnormal or excessive fat accumulation. In recent decades overweight and obesity have become critical public health issues in the world [12], particularly, in low-and middle-income countries [13]. In China, the prevalence of overweight fluctuated between 8.0 and 17.1% for males and between 10.7 and 14.4% for females, respectively; the prevalence of abdominal obesity did between 8.5 and 27.8% for males and between 27.8 and 45.9% for females, respectively, during 1993–2009 [14]. Numerous studies had showed that individuals with abdominal obesity (clinically as central obesity) had greater risk for type 2 diabetes mellitus, hypertension, cardiovascular disease and cancer than those with general obesity [15], and platelet hyper-activation linked to central obesity in addition to promoting oxidative stress and chronic inflammation in the body [16–18]. Waist circumference (WC) as a simple and accurate anthropometric marker of abdominal obesity was easy to perform among subjects [19]. Waist-to-height ratio (WHtR) of a person is defined as the person's waist circumference, divided by the person's height. It has been proposed as an alternative anthropometric index for assessing of central obesity with the exception for effects of age, sex and race. The indicator of adiposity has recently emerged as an important marked to assess risk for cardiometabolic diseases, because it avoided potential influences of confounding factors [20]. Thus, WHtR is thought to be more likely to give an accurate assessment of health, since core body fat (abdominal obesity) is the most dangerous kind of body fat.

Accumulative evidence shows that obesity is associated with oxidative stress and chronic inflammation in the body [4], while hypoxia is a prominent characteristic of adipose tissue caused adipocyte dysfunction during obesity-related inflammation. Hypoxia linked to low oxygen or hypoxia-mediated an inflammatory phenotype in perivascular adipose tissue and a loss of the anticontractile properties [21], and increased in the multaneous consumption of large platelets [22]. Involvement of obesity-related reactive oxygen species generation in the regulations of proliferation, differentiation and maturation of megakaryocytes affected platelet production from megakaryocyte and platelet activation state [23,24]. Weight loss reduced oxidative stress and chronic inflammation in central obesity individuals who had lost about 10% of their initial body weight may be due to reduction of platelet activation and restoration of sensitivity to the physiological antiaggregating agents [18,21], indicating that platelet indices linked to overweight or obesity. The present study aimed to investigate associations between adiposity indices and platelet indices using baseline data from the Dongfeng-Tongji Cohort Study.

## Methods

### Ethics Statement

The study was approved by the Medical Research Ethics Committee of Tongji Medical College, Huazhong University of Science and Technology. All participants provided written informed consent.

### Study population

Among participants (n = 27009) from the Dongfeng-Tongji (DFTJ) Cohort Study [25], 2893 individuals with missing data on the variables (including education, marital status, smoking and drinking status, physical activity, doing housework, values of WC, height, weight, blood pressure, PDW and MPV as well as personal diabetes mellitus, coronary heart disease, stroke and myocardial infarction), as well as 6789 individuals with diabetes mellitus, coronary heart disease, cancer and myocardial infarction were excluded from this study. Finally, a total of 17327 individuals (7677 males and 9650 females) were included in this study.

### Data collection and variables

All participants were interviewed face-to-face with semi-structured questionnaires by the trained interviewers; Data collection included personal socio-demographics (such as age, gender, education and marital status), lifestyle factors (smoking status, passive smoke exposure, drinking status, physical activity, doing housework) and personal medical history. Anthropometric measurements such as height, weight and WC were taken in participants with light indoor clothing and without shoes and physical examination (systolic and diastolic blood pressures as well as heart rate) according to the standard methods during the healthy examination. Body mass index (BMI) was calculated by dividing weight in kilograms by height in meters squared. Additionally, peripheral venous blood samples from individuals were obtained in the vacutainer tubes containing an anticoagulant (ethylenediaminetetraacetic acid and sodium citrate). PDW and MPV were measured using a fully automated analyzer (CELL-DYN 3700, Abbott, USA). The PDW and MPV reference intervals for Chinese adults range from 15.0 to 17.0% and from 7 to 11 femtolitre (fl), respectively [26]. Central obesity was defined as WHtR ≥0.5 or WC ≥90 cm for men and ≥80 cm for women according to the international diabetes foundation criteria published by the Internatdeional Diabetes Federation in 2005 [27,28]. According to body mass index (BMI) classification reference intervals for overweight and obesity Chinese adults, a BMI <24 kg/m^2^ is defined as non-obese, a BMI of 24–27.9 kilogram (kg)/m^2^ as overweight and a BMI of ≥28 kg/m^2^ as obese [29]. Hypertension was defined as a systolic blood pressure >140 mmHg and/or a diastolic blood pressure >90 mmHg, or self-reported hypertension history and taking antihypertensive medicine.

### Statistical analysis

All data from the study participants were anonymized and de-identified prior to the analysis. According to the reference intervals of PDW and MPV for Chinese adults, the participants were classified into low (<15.0% for PDW and <7 fl for MPV), normal (15.0–17.0% for PDW and 7–11 fl for MPV) and high (>17.0% for PDW and >11 fl for MPV) subgroups. To describe the characteristics of the participants, mean with standard deviation and percentage were used for continuous variables and categorical variables, respectively. Chi-square test was used to compare distributions of selected categorical variables. Kruskal-Wallis test was used to compare abnormally distributed data including BMI, systolic and diastolic blood pressures. Multivariable logistic regression models were used to analyze associations between selected variables and PDW or MPV in the subgroups. Participants were classified into the subgroups of males and females by either WCs (males: <90, 90–, 95–, ≥100 cm; females: <80, 80–, 85–, ≥90 cm), or WHtRs (<0.50, 0.50–, 0.55–, ≥0.60) or BMIs (<24, 24–, 26–, ≥28 kg/m^2^). Statistical significance was defined as *p*<0.05. All statistic tests were performed using SPSS 12.0 software (SPSS, Chicago, IL, USA).

## Results

### General characteristics

To judge whether there was a potential sample selection bias in this analysis, the comparisons of basic characteristics between included and excluded populations were conducted prior to the present analysis. The results showed that there was significant differences in certain variables between the included and excluded populations: age, education, smoking status, passive smoke exposure, drinking status and doing housework, although the proportions are close between the populations (*p*<0.01 for all).

As shown in Tables 1 and 2, the mean age of participants was 65.8 years for men and 60.3 years for women (*p*<0.05). With the exception of passive smoke exposure, differences in the proportions of smoking status, drinking status, physical activity, doing housework, WC, WHtR, BMI, MPV, PDW as well as hypertension and hyperlipemia were found between the two sexes (*p*<0.01 for all).

**Table 1:** The socio-demographic and personal characteristics of participants.

Variable		Male (n = 7677)	Female (n = 9650)	Total (n = 17327)	P–value
Age (year, mean±SD)		65.8±6.7	60.3±7.6	62.7±7.7	<0.001^a^
Smoking status (n, %)	Never-smokers	2943 (38.3)	9385 (97.3)	12328 (71.1)	<0.001^b^
	Former smokers	1678 (21.9)	67 (0.7)	1745 (10.1)	
	Current smokers	3056 (39.8)	198 (2.1)	3254 (18.8)	
Passive smoke exposure (yes/no, n, %)		1453 (18.9)/6224 (81.1)	1870 (19.4)/7780 (80.6)	3323 (19.2)/14004 (80.8)	0.453^b^
Drinking status (n, %)	Non-drinker	3725 (48.5)	8918 (92.4)	12643 (73.0)	<0.001^b^
	Former drinker	668 (8.7)	100 (1.0)	768 (4.4)	
	Current drinker	3284 (42.8)	632 (6.5)	3916 (22.6)	
Physical activity (yes/no, n, %)		6862 (89.4)/815 (10.6)	8493 (88.0)/1157 (12.0)	15355 (88.6)/1972 (11.4)	<0.001^b^
Doing housework (yes/no, n, %)		6150 (80.1)/1527 (19.9)	9309 (96.5)/341 (3.5)	15459 (89.2)/1868 (10.8)	0.001^b^

^a^Student’s t–test was used to compare the mean values of continuous variables.

^b^The Chi–square test was used to analyze a relationships between two categorical variables.

**Table 2:** Distributions of clinical variables of participants.

Variable		Male (n = 7677)	Female (n = 9650)	Total (n = 17327)	P–value
**Body mass index (kg/m^2^)**	**<24**	3527 (45.9)	4819 (49.9)	8346 (48.2)	<0.001^a^
	**24–**	1921 (25.0)	2092 (21.7)	4013 (23.2)	
	**26–**	1330 (17.3)	1466 (15.2)	2796 (16.1)	
	**≥28**	899 (11.7)	1272 (13.2)	2171 (12.5)	
**Waist circumference (male/female, cm)**	**<90/<80**	5347 (69.6)	4507 (46.7)	9854 (56.9)	<0.001^a^
	**90–/80–**	434 (5.7)	1659 (17.2)	2093 (12.1)	
	**95–/85–**	1304 (17.0)	2105 (21.8)	3409 (19.7)	
	**≥100/≥90**	592 (7.7)	1379 (14.3)	1971 (11.3)	
**Waist-to-height ratio**	**<0.50**	3256 (42.4)	3952 (41)	7208 (41.6)	<0.001^a^
	**0.50–**	2746 (35.8)	3025 (31.3)	5771 (33.3)	
	**0.55–**	1264 (16.5)	1775 (18.4)	3039 (17.5)	
	**≥0.60**	411 (5.4)	898 (9.3)	1309 (7.6)	
**Mean platelet volume (fl, n, %)**	**Low (<7)**	990 (12.9)	1091 (11.3)	2081 (12.0)	<0.001^a^
	**Normal(7–11)**	6055 (78.9)	7626 (79.0)	13681 (79.0)	
	**High (>11)**	632 (8.2)	933 (9.7)	1565 (9.0)	
**Platelet distribution width (%, n, %)**	**Low (<15)**	2370 (24.6)	1697 (22.1)	4067 (23.5)	<0.001^a^
	**Normal(15–17)**	3601 (37.3)	2669 (34.8)	6270 (36.2)	
	**High (>17)**	3679 (38.1)	3311 (43.1)	6990 (40.3)	
**Hypertension (yes/no, n, %)**		2643 (34.4)/5033 (65.6)	2776 (28.8)/6873 (71.2)	5419 (31.3)/11906(68.7)	<0.001^a^
**Hyperlipemia (yes/no, n, %)**		1312 (17.1)/6364 (82.9)	1452 (15.0)/8197 (85.0)	2764 (16.0)/14561 (84.0)	<0.001^a^

fl = femtolitre.

Subjects were divided into three subgroups according to the reference ranges for Chinese adults: low (<15%), normal (15–17%) and high (>17%) for platelet distribution width value, as well as low (<7 fl), normal (7–11 fl) and high (>11 fl) for mean platelet volume value, respectively.

^a^The Chi–square test was used to analyze a relationships between two categorical variables.

### PDW, MPV and WC

As shown in Table 3, participants were classified into the subgroups based on gender and the WC cut–off points of <90, 90–, 95– and ≥100 cm for males and <80, 80–, 85–, ≥90 cm for females. In the crude model, participants with low MPV or PDW in the highest WC group were at a greater risk of having low MPV (odds ratio (OR): 1.09, 95% confidence interval (CI): 0.94, 1.25, *p*_trend_ = 0.229) or PDW (OR: 1.90, 95%CI: 1.69, 2.13, *p*_trend_<0.001) than those who had low MPV or PDW in the corresponding lowest WC group; participants with high MPV or PDW in the highest WC group were at a lower risk of having high MPV (OR: 0.78, 95%CI: 0.65, 0.94, *p*_trend_ = 0.038) or PDW (OR: 0.68, 95%CI: 0.61, 0.77, *p*_trend_<0.001) than those who had low MPV or PDW in the corresponding lowest WC groups. After adjusting for gender, age, smoking status, passive smoke exposure, drinking status, physical activity, doing housework, hypertension and self-reported hyperlipemia, participants with low MPV or PDW in the highest WC group were at a greater risk of having low MPV (OR: 1.14, 95% CI: 0.98, 1.32, *p*_trend_ = 0.069) or PDW (OR: 2.00, 95% CI: 1.77, 2.27, *p*_trend_<0.001) than those who had low MPV or PDW in the corresponding lowest WC group; participants with high MPV (OR: 0.72, 95% CI: 0.60, 0.87, *p*_trend_ = 0.02) or PDW (OR: 0.68, 95% CI: 0.60, 0.77, *p*_trend_<0.001) in the highest WC group were at a lower risk for having high MPV or PDW than those who had high MPV or PDW in the corresponding lowest WC groups. Both sexes with low PDW in the highest WC groups were at a greater risk of having high PDW (OR: 1.40, 95% CI: 1.09, 1.78 for males, *p*_trend_<0.001; OR: 2.27, 95% CI: 1.96, 2.63 for females, *p*_trend_<0.001) than those who had low PDW in the corresponding lowest WC groups; whereas female participants with high MPV or PDW in the highest WC groups were at a lower risk of having high MPV (OR: 0.70, 95% CI: 0.56, 0.87, *p*_trend_ = 0.003) or PDW (OR: 0.75, 95% CI: 0.65, 0.86, *p*_trend_<0.001) than those who had high MPV or PDW in the corresponding lowest WC groups. Additionally, in the highest WC groups only female participants with low MPV were at a greater risk of having low MPV (OR: 1.26, 95% CI: 1.05, 1.50, *p*_trend_ = 0.004) than those who had low MPV in the corresponding lowest WC groups.

**Table 3:** Association between waist circumstance values and platelet indices among 17327 participants.

Variable			Waist circumstance (cm, OR, 95% CI)				P–trend
**MPV (low/normal)**	**Total**		**<90/<80**	**90–/80–**	**95–/85–**	**≥100/≥90**	
		Univariate model	1.00	1.06 (0.94, 1.19)	1.04 (0.89, 1.21)	1.09 (0.94, 1.25)	0.229
		Multivariate model^a^	1.00	1.08 (0.96, 1.22)	1.07 (0.92, 1.25)	1.14 (0.98, 1.32)	0.069
	**Male**		<90	90–	95–	≥100	
		Univariate model	1.00	1.17 (0.98, 1.39)	1.17 (0.91, 1.49)	0.91 (0.67, 1.24)	0.504
		Multivariate model^b^	1.00	1.16 (0.97, 1.39)	1.15 (0.90, 1.47)	0.88 (0.65, 1.20)	0.701
	**Female**		<80	80–	85–	≥90	
		Univariate model	1.00	1.02 (0.86, 1.21)	1.03 (0.85, 1.25)	1.21 (1.02, 1.44)	0.050
		Multivariate model^b^	1.00	1.04 (0.88, 1.23)	1.06 (0.87, 1.29)	1.26 (1.05, 1.50)	0.020
**MPV (high/normal)**	**Total**		<90/<80	90–/80–	95–/85–	≥100/≥90	
		Univariate model	1.00	0.98 (0.85, 1.12)	1.02 (0.86, 1.21)	0.78 (0.65, 0.94)	0.038
		Multivariate model^a^	1.00	0.94 (0.81, 1.07)	0.96 (0.81, 1.14)	0.72 (0.60, 0.87)	0.003
	**Male**		<90	90–	95–	≥100	
		Univariate model	1.00	0.93 (0.74, 1.17)	0.93 (0.67, 1.27)	0.76 (0.51, 1.12)	0.159
		Multivariate model^b^	1.00	0.94 (0.75, 1.18)	0.94 (0.68, 1.30)	0.76 (0.51, 1.13)	0.186
	**Female**		<80	80–	85–	≥90	
		Univariate model	1.00	0.95 (0.80, 1.13)	0.99 (0.81, 1.21)	0.73 (0.59, 0.90)	0.013
		Multivariate model^b^	1.00	0.93 (0.78, 1.11)	0.96 (0.78, 1.18)	0.70 (0.56, 0.87)	0.004
**PDW (low/normal)**	**Total**		<90/<80	90–/80–	95–/85–	≥100/≥90	
		Univariate model	1.00	1.19 (1.07, 1.32)	1.31 (1.16, 1.48)	1.90 (1.69, 2.13)	<0.001
		Multivariate model^a^	1.00	1.21 (1.09, 1.35)	1.35 (1.19, 1.54)	2.00 (1.77, 2.27)	<0.001
	**Male**		<90	90–	95–	≥100	
		Univariate model	1.00	1.21 (1.03, 1.42)	1.32 (1.07, 1.64)	1.34 (1.05, 1.70)	<0.001
		Multivariate model^b^	1.00	1.23 (1.05, 1.45)	1.36 (1.09, 1.69)	1.40 (1.09, 1.78)	<0.001
	**Female**		<80	80–	85–	≥90	
		Univariate model	1.00	1.20 (1.05, 1.38)	1.36 (1.17, 1.59)	2.19 (1.90, 2.52)	<0.001
		Multivariate model^b^	1.00	1.22 (1.06, 1.40)	1.39 (1.19, 1.63)	2.27 (1.96, 2.63)	<0.001
**PDW (high/normal)**	**Total**		<90/<80	90–/80–	95–/85–	≥100/≥90	
		Univariate model	1.00	0.88 (0.80, 0.96)	0.72 (0.64, 0.81)	0.68 (0.61, 0.77)	<0.001
		Multivariate model^a^	1.00	0.88 (0.80, 0.96)	0.72 (0.64, 0.81)	0.68 (0.60, 0.77)	<0.001
	**Male**		<90	90–	95–	≥100	
		Univariate model	1.00	0.81 (0.71, 0.93)	0.69 (0.57, 0.85)	0.59 (0.47, 0.75)	<0.001
		Multivariate model^b^	1.00	0.78 (0.68, 0.90)	0.67 (0.54, 0.81)	0.56 (0.44, 0.71)	<0.001
	**Female**		<80	80–	85–	≥90	
		Univariate model	1.00	0.97 (0.86, 1.09)	0.79 (0.68, 0.91)	0.77 (0.67, 0.89)	<0.001
		Multivariate model^b^	1.00	0.96 (0.85, 1.08)	0.77 (0.67, 0.89)	0.75 (0.65, 0.86)	<0.001

95%CI: 95% confidence interval; fl = femtolitre; MPV: mean platelet volume; PDW: platelet distribution width; OR: odds ratio.

Subjects were divided into three subgroups according to the reference ranges for Chinese adults: low (<15%), normal (15–17%) and high (>17%) for platelet distribution width value, and mean platelet volume is low (<7 fl), normal (7–11 fl) and high (>11 fl) for mean platelet volume value, respectively.

Subjects were divided into four subgroups according to waist circumstance levels in sex-based groups. The cut-off points of waist circumstance were <90, 90–, 95—and ≥100 cm for male, and <80, 80–, 85—and ≥90 cm for female, respectively.

^a^Adjusted for age (continuous), gender, smoking status, passive smoke exposure, drinking status, physical activity, doing housework, hyperlipemia and hypertension

^b^Adjusted for age (continuous), smoking status, passive smoke exposure, drinking status, physical activity, doing housework, hyperlipemia and hypertension.

### PDW, MPV and WHtR

As shown in Table 4, participants were classified into the subgroups based on gender and the WHtR cut–off points of <0.50, 0.50–, 0.55—and ≥0.60. In the crude model, participants with low MPV or PDW in the highest WHtR groups were at a greater risk of having low MPV (OR: 1.38, 95% CI: 1.17, 1.64, *p*_trend_<0.001) or PDW (OR: 2.32, 95%CI: 2.01, 2.68, *p*_trend_<0.001) than those who had low MPV or PDW in the corresponding lowest WHtR groups; participants with high MPV or PDW in the highest WHtR group were at a lower risk of having high MPV (OR: 0.75, 95% CI: 0.60, 0.94, *p*_trend_ = 0.022) or PDW (OR: 0.64, 95% CI: 0.55, 0.74, *p*_trend_<0.001) than those who had high MPV or PDW in the corresponding lowest WHtR groups. After adjusting for gender, age, marital status, education levels, smoking status, passive smoke exposure, drinking status, physical activity, doing housework, hypertension and self-reported hyperlipemia, in the highest WHtR groups, participants with low MPV or PDW were at a greater risk of having low MPV (OR: 1.42, 95% CI: 1.19, 1.70, *p*_trend_<0.001) or PDW (OR: 2.46, 95% CI: 2.12, 2.85, *p*_trend_<0.001) than those who had low MPV or PDW in the corresponding lowest WHtR groups, whereas participants with high MPV or PDW were at a lower risk of having high MPV (OR: 0.72, 95% CI: 0.57, 0.91, *p*_trend_<0.001) or PDW (OR: 0.61, 95% CI: 0.52, 0.71, *p*_trend_<0.001) than those who had high MPV or PDW in the corresponding lowest WHtR groups, both sexes with low PDW were at a greater risk of having low PDW (OR: 1.97, 95% CI: 1.52, 2.54, *p*_trend_<0.001 for males; OR: 2.82, 95% CI: 2.54, 3.39, *p*_trend_<0.001 for females) than those who had low PDW in the corresponding lowest WHtR groups. In the highest WHtR groups, only female participants with high MPV or PDW were found to be at a lower risk of having high MPV (OR: 0.65, 95% CI: 0.48, 0.87, *p*_trend_ = 0.004) or PDW (OR: 0.65, 95% CI: 0.54, 0.79, *p*_trend_<0.001) compared with those who had high MPV or PDW in the corresponding lowest WHtR groups, whereas, only female participants with low MPV were at a greater risk of having low MPV (OR: 1.68, 95% CI: 1.34, 2.09, *p*_trend_<0.001) than those who had low MPV in the corresponding lowest WHtR groups.

**Table 4:** Association between waist—to—height ratio and platelet indices among 17327 participants.

Variable			Waist-to-height ratio (OR, 95% CI)				P–trend
			<0.50	0.50–	0.55–	≥0.60	
**MPV (low/normal)**	**Total**	Univariate model	1.00	1.06 (0.95, 1.19)	1.24 (1.09, 1.41)	1.38 (1.17, 1.64)	<0.001
		Multivariate model^a^	1.00	1.06 (0.95, 1.18)	1.26 (1.10, 1.43)	1.42 (1.19, 1.70)	<0.001
	**Male**	Univariate model	1.00	1.04 (0.89, 1.22)	1.40 (1.16, 1.68)	1.17 (0.86, 1.58)	0.004
		Multivariate model^b^	1.00	1.04 (0.89, 1.22)	1.37 (1.14, 1.66)	1.12 (0.82, 1.52)	0.011
	**Female**	Univariate model	1.00	1.08 (0.92, 1.25)	1.13 (0.95, 1.36)	1.54 (1.25, 1.90)	<0.001
		Multivariate model^b^	1.00	1.11 (0.95, 1.29)	1.21 (1.00, 1.46)	1.68 (1.34, 2.09)	<0.001
**MPV (high/normal)**	**Total**	Univariate model	1.00	1.02 (0.91, 1.15)	0.91 (0.78, 1.06)	0.75 (0.60, 0.94)	0.022
		Multivariate model^a^	1.00	1.02 (0.90, 1.15)	0.89 (0.76, 1.04)	0.72 (0.57, 0.91)	<0.001
	**Male**	Univariate model	1.00	1.06 (0.88, 1.27)	0.93 (0.73, 1.20)	0.86 (0.58, 1.28)	0.495
		Multivariate model^b^	1.00	1.06 (0.88, 1.27)	0.95 (0.74, 1.22)	0.86 (0.58, 1.30)	0.557
	**Female**	Univariate model	1.00	1.00 (0.86, 1.18)	0.88 (0.73, 1.07)	0.68 (0.51, 0.90)	0.011
		Multivariate model^b^	1.00	0.98 (0.84, 1.15)	0.84 (0.69, 1.03)	0.65 (0.48, 0.87)	0.004
**PDW (low/normal)**	**Total**	Univariate model	1.00	1.20 (1.09, 1.32)	1.48 (1.33, 1.65)	2.32 (2.01, 2.68)	<0.001
		Multivariate model^a^	1.00	1.22 (1.11, 1.34)	1.53 (1.36, 1.71)	2.46 (2.12, 2.85)	<0.001
	**Male**	Univariate model	1.00	1.15 (1.00, 1.33)	1.32 (1.12, 1.56)	1.86 (1.45, 2.39)	<0.001
		Multivariate model^b^	1.00	1.17 (1.01, 1.35)	1.37 (1.15, 1.62)	1.97 (1.52, 2.54)	<0.001
	**Female**	Univariate model	1.00	1.23 (1.09, 1.40)	1.61 (1.39, 1.86)	2.60 (2.18, 3.10)	<0.001
		Multivariate model^b^	1.00	1.27 (1.11, 1.44)	1.69 (1.46, 1.97)	2.82 (2.34, 3.39)	<0.001
**PDW (high/normal)**	**Total**	Univariate model	1.00	0.93 (0.86, 1.00)	0.72 (0.66, 0.80)	0.64 (0.55, 0.74)	<0.001
		Multivariate model^a^	1.00	0.89 (0.83, 0.97)	0.69 (0.62, 0.76)	0.61 (0.52, 0.71)	<0.001
	**Male**	Univariate model	1.00	0.93 (0.83, 1.04)	0.62 (0.53, 0.72)	0.59 (0.46, 0.76)	<0.001
		Multivariate model^b^	1.00	0.89 (0.79, 1.00)	0.57 (0.49, 0.67)	0.54 (0.42, 0.70)	<0.001
	**Female**	Univariate model	1.00	0.91 (0.82, 1.01)	0.82 (0.72, 0.94)	0.69 (0.57, 0.84)	<0.001
		Multivariate model^b^	1.000	0.89 (0.80, 0.99)	0.79 (0.68, 0.90)	0.65 (0.54, 0.79)	<0.001

95%CI: 95% confidence interval; fl = femtolitre; MPV: mean platelet volume; PDW: platelet distribution width; OR: odds ratio.

Subjects were divided into three subgroups according to the reference ranges for Chinese adults: low (<15%), normal (15–17%) and high (>17%) for platelet distribution width, as well as low (<7 fl), normal (7–11 fl), high (>11 fl) for mean platelet volume, respectively.

The subjects were divided into four groups according to waist—to—height ratios: <0.50, 0.50–, 0.55—and ≥0.60.

^a^Adjusted for age (continuous), gender, smoking status, passive smoke exposure, drinking status, physical activity, doing housework, hyperlipemia and hypertension.

^b^Adjusted for age (continuous), smoking status, passive smoke exposure, drinking status, physical activity, doing housework, hyperlipemia and hypertension.

### PDW, MPV and BMI

As shown in Table 5, participants were classified into two subgroups based on gender and the BMI cut–off points of <24, 24–, 26–, and ≥28 kg/m^2^. In crude model, in the highest BMI group, participants with low MPV or PDW were at a greater risk of having low MPV (OR: 1.21, 95% CI: 1.05, 1.39, *p*_trend_ = 0.002) or PDW (OR: 1.25, 95% CI: 1.10, 1.41, *p*_trend_<0.001) than those who had low MPV or PDW in the corresponding lowest BMI group, but participants with high PDW were at a greater risk for having high PDW (OR: 1.34, 95% CI: 1.14, 1.57, *p*_trend_<0.001) than those who had high PDW in the corresponding the lowest BMI group. No significant change was observed, after adjusting for gender, age, marital status, education levels, smoking status, passive smoke exposure, drinking status, physical activity, doing housework, hypertension and self-reported hyperlipemia. In the highest BMI groups only females with low MPV or PDW were at a greater risk for having low MPV (OR: 1.33, 95% CI: 1.10, 1.62, *p*_trend_<0.001) or PDW (OR: 1.34, 95% CI: 1.14, 1.58, *p*_trend_<0.001) than those who had low MPV in the corresponding lowest BMI groups.

**Table 5:** Association between body index mass and platelet indices among 17327 participants.

Variable			Body mass index (kg/m^2^, OR, 95% CI))				P–trend
			<24	24–	26–	≥28	
**MPV (low/normal)**	**Total**	Univariate model	1.00	0.94 (0.83, 1.06)	1.17 (1.03, 1.33)	1.21 (1.05, 1.39)	0.002
		Multivariate model^a^	1.00	0.93 (0.82, 1.04)	1.16 (1.01, 1.32)	1.20 (1.04, 1.39)	0.004
	**Male**	Univariate model	1.00	0.83 (0.70, 0.98)	1.04 (0.86, 1.25)	1.10 (0.89, 1.36)	0.420
		Multivariate model^b^	1.00	0.83 (0.70, 0.99)	1.05 (0.87, 1.27)	1.08 (0.87, 1.35)	0.456
	**Female**	Univariate model	1.00	1.03 (0.87, 1.22)	1.28 (1.07, 1.53)	1.31 (1.08, 1.58)	0.001
		Multivariate model^b^	1.00	1.04 (0.88, 1.23)	1.30 (1.08, 1.56)	1.33 (1.10, 1.62)	<0.001
**MPV (high/normal)**	**Total**	Univariate model	1.00	1.02 (0.89, 1.16)	1.00 (0.86, 1.17)	1.08 (0.92, 1.28)	0.447
		Multivariate model^a^	1.00	1.02 (0.89, 1.16)	1.00 (0.85, 1.16)	1.07 (0.91, 1.27)	0.523
	**Male**	Univariate model	1.00	1.03 (0.84, 1.26)	1.10 (0.87, 1.38)	1.23 (0.95, 1.59)	0.123
		Multivariate model^b^	1.00	1.01 (0.82, 1.24)	1.10 (0.87, 1.40)	1.23 (0.95, 1.61)	0.119
	**Female**	Univariate model	1.00	1.04 (0.87, 1.25)	0.95 (0.78, 1.17)	1.00 (0.81, 1.23)	0.845
		Multivariate model^b^	1.00	1.02 (0.85, 1.21)	0.93 (0.75, 1.14)	0.98 (0.79, 1.22)	0.647
**PDW (low/normal)**	**Total**	Univariate model	1.00	1.07 (0.97, 1.18)	1.14 (1.02, 1.28)	1.25 (1.10, 1.41)	<0.001
		Multivariate model^a^	1.00	1.06 (0.96, 1.18)	1.14 (1.02, 1.28)	1.26 (1.11, 1.43)	<0.001
	**Male**	Univariate model	1.00	1.00 (0.86, 1.16)	0.98 (0.83, 1.17)	1.13 (0.92, 1.37)	0.429
		Multivariate model^b^	1.00	1.00 (0.86, 1.17)	1.00 (0.84, 1.19)	1.16 (0.94, 1.42)	0.615
	**Female**	Univariate model	1.00	1.12 (0.98, 1.29)	1.28 (1.10, 1.49)	1.34 (1.14, 1.57)	<0.001
		Multivariate model^b^	1.00	1.12 (0.97, 1.28)	1.26 (1.08, 1.47)	1.34 (1.14, 1.58)	<0.001
**PDW (high/normal)**	**Total**	Univariate model	1.00	1.07 (0.98, 1.17)	1.08 (0.98, 1.19)	1.15 (1.03, 1.28)	0.006
		Multivariate model^a^	1.00	1.04 (0.95, 1.14)	1.05 (0.95, 1.16)	1.13 (1.01, 1.26)	0.043
	**Male**	Univariate model	1.00	1.06 (0.94, 1.21)	1.07 (0.93, 1.24)	1.11 (0.94, 1.32)	0.150
		Multivariate model^b^	1.00	1.02 (0.90, 1.16)	1.02 (0.88, 1.18)	1.05 (0.88, 1.24)	0.299
	**Female**	Univariate model	1.00	1.05 (0.93, 1.18)	1.06 (0.93, 1.22)	1.18 (1.02, 1.36)	0.028
		Multivariate model^b^	1.00	1.06 (0.94, 1.19)	1.07 (0.93, 1.22)	1.19 (1.02, 1.37)	0.027

95%CI: 95% confidence interval; fl = femtolitre; MPV: mean platelet volume; PDW: platelet distribution width; OR: odds ratio.

Subjects were divided into three subgroups according to the reference ranges for Chinese adults: low (<15%), normal (15–17%) and high (>17%) for platelet distribution width value, as well as low (<7 fl), normal (7–11 fl) and high (>11 fl) for mean platelet volume value, respectively.

The subjects were divided into four groups according to body mass index: <24 kg/m2, 24 kg/m2–, 26 kg/m2–, ≥28 kg/m2.

^a^Adjusted for age (continuous), gender, smoking status, passive smoke exposure, drinking status, physical activity, doing housework, hyperlipemia and hypertension.

^b^Adjusted for age (continuous), smoking status, passive smoke exposure, drinking status, physical activity, doing housework, hyperlipemia and hypertension.

## Discussion

Previous studies had reported that MPV and PDW could predict activation of coagulation more efficiently [1], moreover, central obesity (the principal symptom of metabolic syndrome) individuals could be at higher risk of obesity-related chronic diseases compared with those who had general obesity [15], moreover, MPV was inversely associated with metabolic syndrome [30]. The findings showed that participants with higher BMI were at a greater risk of having low MPV and PDW values, and participants with higher WHtR or WC had abnormal MPV and PDW values, implying that WHtR or WC may be a sensitive indicator for reflecting obesity-related chronic diseases in relation to abnormal platelet activity. Further the results from the multiple linear regression analysis indicated that in female participants, the values of MPV and PDW were reduced 1.9% amd 2.9%, every increase in BMI of 10kg/m^2^, 1.3% and 3.6% every increase in WC of 10cm and 2.4% and 6.3% every increase in WHtR of 10% (data not shown).

It is well known that platelet mass (platelet count × MPV) is kept constant, while MPV was inversely related to platelet counts [31], both PDW and MPV usually changed in the same direction [1]. As participants with certain diseases including diabetes mellitus, coronary heart disease, cancer and myocardial infarction in relation to MPV and PDW values were excluded from the study, obesity in relation to reduced MPV and PDW values may attribute to a physiological change of platelet counts.

Multiple studies suggested that reduced MPV and PDW may result from induced inflammation involved in certain diseases such as mild cognitive impairment and AD [3]. Multiple factors including Interleukin-1 alpha, interleukin-3, epidermal growth factor, tumor necrosis factor-alpha and granulocyte colony-stimulating factor had a high accuracy (96%) to predict clinical AD [32]. In this study, more than 90% of the participants aged 45 and over, which may contribute to increase risk of overweight or obesity, because increased obesity among the middle and old aged people (40–59 years) was obviously contributed to the prevalences of AD and dementia [33]. Thus, the possible reasons for reduced MPV and PDW relating to inflammation may be that adiposity tissue secreted a variety of adipokines, cytokines (such as leptin, adiponectin, interleukin-6, interleukin-1, and tumor necrosis factor- alpha) and pro-inflammatory cytokines, which could lead to chronic low—grade inflammation [16]. Furthermore, reduced MPV and PDW were related to increased consumption of large platelets, which was probably due to vasculature inflammation [2,22]. In obese individuals, adipose tissue hypoxia may induce dysregulate adipocytokines production involved in excessive ROS generation and vascular inflammation [21,23,24]. A few studies have recently reported that hypoxia and ROS were related to platelet activation or platelet generation and thereby may affect proliferation and differentiation of megakaryocytes [24,34]. Platelet formation is the consequence of caspase activation within megakaryocytes [24]. Chronic hypoxia accelerated the proliferation and differentiation of megakaryocytes in bone marrow and increased their functional activity [34], probably thereby reduced MPV and PDW values. Moreover, ROS increased platelet activation and aggregation with vascular endothelial layer damaged and caused increase in impaired production of platelets, whereas, low MPV was associated with impaired production rather than increased destruction of platelet [35]. Our results indicated that obesity individuals had an obvious abnormal change in PDW rather than MPV, in addition to PDW as a reliable indicator to identify hyperdestructive and hypoproductive thrombocytopenia [36]. With the addition of certain diseases relating to abnormal MPV and PDW, genetic factor was also a major determinant for MPV and PDW values, because genetic variation accounted for 69.0% for MPV and 34% for PDW, respectively [37].

The combined measurement of BMI and WC is the simple and inexpensive anthropometric measurement. It is well known that physical indices of obesity related to risk of certain diseases including hypertension, type-2 diabetes, and/or dyslipidema. A previous study reported that BMI and WC are important predictors for obesity in Chinese in addition to WHtR [38]. Additionally, studies showed that mean body weight has increased dramatically in older people either in Western conturies or in Asia [39], whereas increased central body fat was inversely associated with bone mineral density relating to reduced PDW and MPV values [5,40]. Thus, weight is one of osteoporosis risk factors in obese people. However, we did not know whether there is difference in adverse biological effects in response to these indices between both sexes. The present study implied that females with higher BMI (greater than or equal to 26) seem to be at greater risk for abnormal values of platelet indices rather than males, which may link to potential differences in metabolic, behavioral and psychosocial factors between both sexes. WHtR or WC is more accuracy than BMI in response to adipose tissue accumulation in the upper body (abdominal obesity). We found strong associations between WC, WHtR or BMI and abnormal MPV or PDW among women rather than men. A possible reason for gender differences in obesity is that Chinese women may tend to have a higher fat percentage than Chinese men [41]. Furthermore, abdominal obesity in Chinese adults has been given greater concern rather than general obesity. Changes in dietary patterns, nutritional status and physical activity may be the major reasons for increased risk for general overweight, obesity and abdominal obesity in Chinese adults [14,42]. Thus, public health prevention strategies in China are required to modify health behaviors in order to control obesity-related outcomes, to decrease risk for obesity-related diseases including AD, mild cognitive impairment and dementia [33,43].

The limitations of the present study are as follows: firstly, the results were based on the baseline data of a cohort study, the causal causal links between adiposity indices with PDW and MPV need be further evaluated; secondly, dietary habits might influence serum MPV and PDW values but was not considered as a confounding factor, considering that participants have lived the same city for more than several decades and have a relatively homogenous trend in their dietary habits; finally, the exclude individuals with certain self-reported diseases may induce select bias between included and excluded individuals, owing to their unhealthy lifestyles changes, for instance, the percntages of smokers (18.8%) and drinkers (22.6%) in the excluded individuals were higher than those in former smokers (16.3%) and drinkers (17.7%) in the included ones (data not shown). Thus, it should be cautious for us to explain the results.

## Conclusions

WC and WHtR were more sensitive rather than BMI to reflect changes of MPV and PDW values, the pattern of change in WC and WHtR may imply the occurrence of subclinical symptoms. The females with high BMI values along with reduced MPV or PDW values may have a potential risk of developing certain diseases.
